# Blood biomarkers with Parkinson's disease clusters and prognosis: The oxford discovery cohort

**DOI:** 10.1002/mds.27888

**Published:** 2019-11-06

**Authors:** Michael Lawton, Fahd Baig, Greg Toulson, Alireza Morovat, Samuel G. Evetts, Yoav Ben‐Shlomo, Michele T. Hu

**Affiliations:** ^1^ Population Health Sciences, Bristol Medical School University of Bristol Bristol UK; ^2^ Nuffield Department of Clinical Neurosciences, Division of Clinical Neurology University of Oxford Oxford UK; ^3^ Oxford Parkinson's Disease Centre University of Oxford Oxford UK; ^4^ Department of Clinical Biochemistry Oxford University Hospitals NHS Foundation Trust Oxford UK; ^5^ Department of Clinical Neurology Oxford University Hospitals NHS Foundation Trust Oxford UK

**Keywords:** Parkinson's disease, cohort studies, prognosis, blood biomarkers

## Abstract

**Background:**

Predicting prognosis in Parkinson's disease (PD) has important implications for individual prognostication and clinical trials design and targeting novel treatments. Blood biomarkers could help in this endeavor.

**Methods:**

We identified 4 blood biomarkers that might predict prognosis: apolipoprotein A1, C‐reactive protein, uric acid and vitamin D. These biomarkers were measured in baseline serum from 624 Parkinson's disease subjects (median disease duration, 1.0 years; interquartile range, 0.5–2.0) from the Oxford Discovery prospective cohort. We compared these biomarkers against PD subtypes derived from clinical features in the baseline cohort using data‐driven approaches. We used multilevel models with MDS‐UPDRS parts I, II, and III and Montreal Cognitive Assessment as outcomes to test whether the biomarkers predicted subsequent progression in motor and nonmotor domains. We compared the biomarkers against age of PD onset and age at diagnosis. The q value, a false‐discovery rate alternative to *P* values, was calculated as an adjustment for multiple comparisons.

**Results:**

Apolipoprotein A1 and C‐reactive protein levels differed across our PD subtypes, with severe motor disease phenotype, poor psychological well‐being, and poor sleep subtype having reduced apolipoprotein A1 and higher C‐reactive protein levels. Reduced apolipoprotein A1, higher C‐reactive protein, and reduced vitamin D were associated with worse baseline activities of daily living (MDS‐UPDRS II).

**Conclusion:**

Baseline clinical subtyping identified a pro‐inflammatory biomarker profile significantly associated with a severe motor/nonmotor disease phenotype, lending biological validity to subtyping approaches. No blood biomarker predicted motor or nonmotor prognosis. © 2019 The Authors. *Movement Disorders* published by Wiley Periodicals, Inc. on behalf of International Parkinson and Movement Disorder Society.

## Introduction

Parkinson's disease (PD) is a heterogeneous multisystem neurodegenerative disorder displaying marked variation in phenotype and individual prognosis. There is a critical need for biomarkers to predict both motor and nonmotor outcomes to guide research into disease mechanisms, maximize power in clinical trials, and delineate subtypes. The ultimate aim would be to use this information to identify patients at risk of early deterioration for treatment with drugs aimed at slowing or halting the disease process. In clinical practice, this would have significant implications for the individual (personalized treatment and future planning), as well as directing health and social service resource use.

A blood‐based biomarker, particularly if commercially available, would confer a number of advantages including ease of sample collection, cost, and scalability. We selected 4 potential blood‐based biomarkers based on expert opinion and contemporary evidence^1^, ^2^: vitamin D, apolipoprotein A1 (ApoA1), uric acid, and C‐reactive protein (CRP).

There is high‐quality and reproducible evidence that vitamin D is lower in PD cases compared with controls.^3^, ^4^, ^5^ The mechanisms underpinning this association are not clear, but evidence for a causal role comes from preclinical studies showing a neuroprotective effect in animal models^6^ and the potential association of vitamin D receptor (VDR) polymorphisms with PD, although the studies to date have been inconsistent.^7^, ^8^, ^9^

Higher levels of ApoA1 may confer some form of neuroprotection. This was identified as a protein of interest by a screening method for candidate plasma proteins in a cohort study, and lower levels of ApoA1 were associated with an earlier age at PD onset.^10^ They also reported lower ApoA1 correlating with dopamine transporter (DAT) deficit using an enriched cohort of people at high risk of developing PD.^10^ Mechanisms by which circulating ApoA1 levels may affect progression are speculative but may include an immunomodulatory effect^11^ or even its effect on cardiovascular or cerebrovascular risk.^12^, ^13^, ^14^

Lower uric acid levels have been linked with a higher risk of developing PD in large prospective incidence studies,^15^, ^16^, ^17^, ^18^, ^19^ with lower levels associated with worsening disease severity^20^ and a greater rate of decline.^21^, ^22^, ^23^ The evidence for uric acid being neuroprotective could relate to the role of uric acid as an antioxidant and free‐radical scavenger, which may reduce the oxidative stress causing dopaminergic loss in PD.^24^, ^25^

Evidence for the role of the inflammation in PD is underpinned by postmortem studies,^25^, ^26^, ^27^ animal models,^28^ and positron emission topography imaging in vivo.^29^ A proposed mechanism by which this pathway may accelerate neurodegeneration is that once the microglia are primed, they may subsequently have an exaggerated response to systemic inflammation, increasing neuronal damage.^30^

More recent evidence also suggests that CRP alongside other markers of a proinflammatory state might predict prognosis in incident PD.^31^

The use of a large early‐PD cohort of well‐characterized patients in a longitudinal study, such as the Oxford Discovery cohort, would allow rigorous external evaluation of these baseline biomarkers as well as testing for supportive evidence of biological validity to underpin the Parkinson's clinical subtypes we have previously reported in more than 2500 early‐PD patients recruited from the Oxford Discovery and Tracking Parkinson's longitudinal cohorts,^32^, ^33^

## Materials and Methods

### Patient Selection With Inclusion/Exclusion Criteria

PD patients diagnosed within the past 3.5 years were prospectively recruited as part of the Oxford Parkinson's Disease Centre Discovery cohort study from 11 hospitals across the Thames Valley covering a population of approximately 2.1 million (PD‐Discovery website: http://opdc.medsci.ox.ac.uk). Full details of this cohort are described elsewhere,^34^ with participants recruited between September 2010 and January 2015. We only included individuals in this study if they had a probability of PD ≥ 90% as rated by a research neurologist/movement disorder specialist at the latest longitudinal follow‐up visit. Patients were followed up in clinic every 18 months. Some patients who found it too difficult to attend clinic converted to telephone follow‐up, still every 18 months.

### Patient Evaluation

Assessments of patients was via self‐completed questionnaires and from outpatient clinics using standardized and validated scales both at baseline and follow‐up. For this article we focused on 4 outcome measures: motor function as measured by the Movement Disorders Society (MDS) revised Unified Parkinson's Disease Rating Scale (UPDRS) part III, activities of daily living as measured by the MDS‐UPDRS part II, nonmotor aspects as measured by the MDS‐UPDRS part I, and cognition as measured by the Montreal Cognitive Assessment (MoCA) adjusted for education years. Only MDS‐UPDRS parts I and II were available for those patients who converted to telephone follow‐up.

We also used data‐derived PD subtypes from a range of motor, nonmotor, and cognitive symptoms at baseline. These clusters were calculated using a factor analysis followed by a k‐means cluster analysis considering 2 to 5 clusters. In our first article on this subject we used only the Discovery cohort (n = 769) and decided that 5 clusters gave us the optimal solution.^32^ In our second article on this subject we used 2 cohorts, with the Tracking Parkinson's cohort (n = 1601) chosen to be the development cohort (as it was larger) and the Discovery cohort (n = 944) chosen to be the validation cohort.^33^ In this article we decided that 4 clusters gave us the optimal solution. Comparing the actual and predicted clusters in Discovery gave us a kappa statistic of 0.58, indicating moderate agreement and providing evidence our cluster approach was stable across the 2 cohorts. These 4 clusters were shown to be associated with subsequent motor progression over an average of 3 years and also with medication response using a levodopa challenge. The identified clusters were named (1) fast motor progression with symmetrical motor disease, poor olfaction, cognition, and postural hypotension; (2) mild motor and nonmotor disease with intermediate motor progression; (3) severe motor disease, poor psychological well‐being, and poor sleep with an intermediate motor progression; and (4) slow motor progression with tremor‐dominant, unilateral disease. In this article we compare our biomarkers against the 4 clusters from our development/validation article.^33^

The laboratory assays were carried out by the use of Abbott reagents on their autoanalysers (Abbott Diagnostics, Chicago, IL) according to the manufacturer's instructions. Serum CRP and ApoA1 were measured using immunotubidometric assays on an Architect C16000, as described in kit inserts 8G65‐21 30‐4143/R1 and 9D92‐21 3‐4624/R02, respectively. Serum uric acid was measured using a linked enzymatic assay, also on an Architect C16000, as described in kit insert 3P39 304647/R02. Serum 25(OH)‐VitD was measured using a 1‐step immunoassay on an Architect i2000, as described in kit insert 3L52 49‐8941/R02.

For more in‐depth details of how we used a patient's baseline serum to measure the 4 blood biomarkers, see the web appendix.

### Standard Protocol Approvals, Registrations, and Patient Consents

The study was undertaken with the understanding and written informed consent of each subject, with the approval of the local National Health Service ethics committee, and in compliance with national legislation and the Declaration of Helsinki.

### Statistical Analysis

We decided that we would normalize all our biomarkers, which would allow someone to more easily compare one biomarker against another. After any transformation we tested for normality using a Kolmogorov‐Smirnov (KS) test, for which a significant *P* value would show departure from a normal distribution.

We compared these biomarkers against our data‐derived clinical clusters using multinomial logistic regression models (with the clusters as the outcome) adjusted for age, sex, and disease duration from diagnosis.

Our 4 prognostic outcomes of interest (MDS‐UPDRS parts I, II, and III and MoCA) were modeled longitudinally using multilevel (random slope and intercept) models in which the time axis was time since diagnosis in years. We adjusted all our longitudinal models for age at diagnosis and sex, which were associated with both intercept (baseline value) and slope (rate of change); see web Figure 1 for guide to interpretation. As a sensitivity analysis we used pattern‐mixture models that adjust for those dropping out of the study, as we were concerned that dropout could bias our estimates.^35^ Also as a sensitivity analysis we imputed missing data using the mean score of the answered questions if 80% or more of the questions were answered in the 4 outcomes. We carried out another sensitivity analysis in which we adjusted MDS‐UPDRS parts II and III for the levodopa‐equivalent daily dose. Precise details of how we did this are in the web appendix. Finally, we compared these biomarkers against age at diagnosis and age at onset using regression models adjusted for sex and disease duration.

We used a false discovery rate (FDR) method,^36^ often called the Benjamini‐Hochberg method, to control for multiple comparisons. This method is not quite as strict as the more common familywise error rate Bonferroni correction. We derived q values when we hoped to find a q < 0.05, if that was our significance threshold. Because these q values do not have a direct probabilistic interpretation like *P* values, it is only important whether they reach the significance threshold. An FDR of 5% using q values would mean that 5% of results called significant are false‐positives. We considered the clinical cluster, age, and each prognosis analysis to be completely separate analyses when implementing this method, so only 4 *P* values are entered into this method at a time. As well as reporting the q values, we still report the raw *P* values so that if someone disagrees with our method they could implement their own.

### Computing

All our analyses were carried out in STATA 15 except the pattern‐mixture models, which were computed in MPlus 8.

## Results

A total of 624 PD patients had their baseline serum analyzed (see web Fig. 2 to see how we arrived at that number). The cohort was mostly male (65%), with an average age at diagnosis of 66 years (Table 1). At baseline the median disease duration from diagnosis was 1.0 years, and the median levodopa‐equivalent daily dose (LEDD) at baseline was 300 mg. Only 11.9% of patients were untreated at baseline, 56.4% were on levodopa, and 29.5% were on a dopamine agonist.

**Table 1:** Baseline demographics of the 624 PD patients

Variable	Mean (SD); median (IQR) or n (%)
Sex (female)	219 (35.1%)
Age (years)	67.6 (10.0); 69.1 (61.1 to 74.9)
Age at diagnosis (years)	66.3 (10.1); 67.7 (59.6 to 73.8)
Disease duration from diagnosis (years)	1.3 (0.96); 1.0 (0.5 to 2.0)
LEDD total (mg)	288 (212); 300 (100 to 400)
Untreated	74 (11.9%)
Levodopa use	352 (56.4%)
Dopamine agonist use	184 (29.5%)
MDS‐UPDRS III	26.3 (11.3); 25 (18 to 34)
MoCA	24.7 (3.4); 25 (23 to 27)
MDS‐UPDRS II	8.8 (6.2); 8 (4 to 12)
MDS‐UPDRS I	8.9 (5.3); 8 (5 to 12)
ApoA1 (g/L)	1.6 (0.3); 1.6 (1.4 to 1.8)
CRP (mg/L)	2.9 (7.1); 1.3 (0.6 to 2.5)
Uric acid (umol/L)	307 (76); 307 (253 to 358)
Vitamin D (nmol/L)	49.1 (25.0); 46 (31.1 to 62.5)

LEDD, levodopa‐equivalent daily dose; MDS‐UPDRS, Movement Disorder Society Unified Parkinson's Disease Rating Scale; MoCA, Montreal Cognitive Assessment; ApoA1, apolipoprotein A1; CRP, C‐reactive protein.

The average follow‐up time for clinic visits was 3.2 ± 1.9 years, and when including telephone visits it was 3.5 years. Web Table 1 shows the proportion with data at each follow‐up visit (a maximum of 5 visits), the number withdrawn at each point, and the amount of outcome data we had available at each visit. The number of individuals withdrawing from our study is consistent with other longitudinal studies at 10%–12% (depending on whether it is clinic or telephone follow‐up) withdrawing after the baseline visit and 31%–36% having withdrawn by visit 5 after 6 years of follow‐up.

### Normalizing Blood Biomarker Data

ApoA1 level looked normally distributed, so it was just standardized to unit standard deviation (KS *P* = 0.66). For CRP we first log‐transformed the variable and then standardized it to unit standard deviation (KS *P* = 0.20), similar to a previous study.^37^ Uric acid level looked normally distributed, and because there is a profound sex effect on uric acid, we standardized sex (KS for men, *P* = 0.62; KS for women, *P* = 0.22). As vitamin D varies by season, we standardized the raw data using the methods described by Lawlor et al,^38^ which involved log‐transforming the variable and then adjusting for seasonal variation using a linear regression model with a trigonometric function (KS *P* = 0.10).

### Biomarker Versus Data‐Derived PD Subtypes

When considering our PD subtypes (clusters) against our biomarkers (see Table 2), we found strong differences in ApoA1 (*P* < 0.001) and CRP (*P* = 0.02) levels. In both biomarkers associations seem to be driven by the third cluster, which was our severe motor, poor psychological well‐being, and poor sleep cluster.^33^ This cluster had lower ApoA1 and higher CRP levels. These differences remained significant after adjusting for multiple comparisons (ApoA1, q = 0.001; CRP, q = 0.03).

**Table 2:** Biomarkers versus clusters from PD subtypes article, adjusted for age, sex, and disease duration

Blood biomarker	Cluster 1 n = 210	Cluster 2 n = 104	Cluster 3 n = 152	Cluster 4 n = 155	Adjusted*P* value	q value
ApoA1 (g/L)	1.63 (0.24)	1.69 (0.25)	1.53 (0.22)	1.64 (0.27)	< 0.001	0.001
CRP (mg/L)	2.65 (8.1)	1.93 (2.7)	4.06 (9.7)	2.6 (4.1)	0.02	0.03
Uric acid^a^ (umol/L)	306 (75)	292 (67)	323 (77)	304 (81)	0.22	0.30
Vitamin D (nmol/L)	48.0 (22)	51.4 (27)	46.8 (28)	50.6 (24)	0.38	0.38

ApoA1, apolipoprotein A1; CRP, C‐reactive protein.

a Uric acid was standardised by sex so the adjusted associations are not adjusted with a sex term in the model.

### Longitudinal Analysis

#### Motor predictors

The adjusted results from our main analysis are in Table 3, whereas the crude results are in web Table 2. There was a modest association between uric acid and MDS‐UPDRS III (*P* = 0.03), with a 1 standard deviation increase associated with a 0.36 increase in MDS‐UPDRS III change per year. Hence, raised uric acid was associated with worse motor prognosis. However this association was not significant after adjusting for multiple comparisons (q = 0.11).

**Table 3:** Longitudinal follow‐up associations (per standard deviation change in transformed biomarker)

	Adjusted associations			
MDS‐UPDRS III	Intercept	Slope (per year)	Intercept q value	Slope q value
ApoA1	−0.90 (−2.06 to 0.25); 0.12	−0.06 (−0.40 to 0.29); 0.74	0.17	0.91
CRP	−0.35 (−1.39 to 0.69); 0.51	0.26 (−0.05 to 0.57); 0.09	0.51	0.19
Uric acid^a^	−0.96 (−2.02 to 0.10); 0.08	0.36 (0.04 to 0.68); 0.03	0.17	0.11
Vitamin D	−0.82 (−1.87 to 0.24); 0.13	0.02 (−0.29 to 0.33); 0.91	0.17	0.91
MoCA	Adjusted associations			
	Intercept	Slope (per year)	Intercept q value	Slope q value
ApoA1	0.29 (−0.02 to 0.60); 0.07	0.00 (−0.08 to 0.08); 0.99	0.24	0.99
CRP	−0.19 (−0.48 to 0.09); 0.18	−0.02 (−0.10 to 0.05); 0.50	0.24	0.67
Uric acid^a^	0.21 (−0.08 to 0.50); 0.16	−0.03 (−0.10 to 0.05); 0.48	0.24	0.67
Vitamin D	0.16 (−0.13 to 0.45); 0.27	0.03 (−0.05 to 0.10); 0.48	0.27	0.67
MDS‐UPDRS II	Adjusted associations			
	Intercept	Slope (per year)	Intercept q value	Slope q value
ApoA1	−0.93 (−1.50 to −0.35); 0.002	−0.07 (−0.21 to 0.07); 0.34	0.004	0.45
CRP	0.81 (0.29 to 1.34); 0.002	0.11 (−0.03 to 0.24); 0.12	0.004	0.29
Uric acid^a^	0.04 (−0.49 to 0.57); 0.88	0.04 (−0.09 to 0.18); 0.53	0.88	0.53
Vitamin D	−0.75 (−1.28 to −0.22); 0.005	−0.10 (−0.23 to 0.03); 0.14	0.007	0.29
MDS‐UPDRS I	Adjusted Associations			
	Intercept	Slope (per year)	Intercept q value	Slope q value
ApoA1	−0.82 (−1.31 to −0.33); <0.001	−0.02 (−0.13 to 0.09); 0.76	0.004	0.76
CRP	0.45 (0.01 to 0.90); 0.047	0.06 (−0.04 to 0.17); 0.22	0.06	0.61
Uric acid^a^	0.53 (0.08 to 0.99); 0.02	−0.05 (−0.16 to 0.05); 0.31	0.04	0.61
Vitamin D	−0.41 (−0.86 to 0.05); 0.08	−0.03 (−0.13 to 0.07); 0.56	0.08	0.75

MDS‐UPDRS, Movement Disorder Society Unified Parkinson's Disease Rating Scale; MoCA, Montreal Cognitive Assessment; ApoA1, apolipoprotein A1; CRP, C‐reactive protein.

Data (except where stated) are estimate (95% confidence interval); *P* value. Models are adjusted for age at diagnosis and sex.

a Uric acid was standardized by sex so the adjusted associations are not adjusted with a sex term in the model.

#### Cognitive predictors

There were no strong associations between the biomarkers with either MoCA intercept or slope.

#### Activities of daily living predictors

ApoA1, CRP, and vitamin D were all strongly associated with the MDS‐UPDRS II intercept, both before (*P* < 0.05) and after adjustment for multiple comparisons (q < 0.05). Hence, reduced ApoA1, raised CRP, and reduced vitamin D were associated with worse activities of daily functionality at baseline.

#### Nonmotor predictors

ApoA1, CRP, and uric acid were associated with the MDS‐UPDRS I intercept (*P* < 0.05). Hence, reduced ApoA1, raised CRP and raised uric acid were associated with worse nonmotor function at baseline. ApoA1 (q = 0.004) and uric acid (q = 0.04) remained significant after adjusting for multiple comparisons; however, CRP was no longer significant (q = 0.06).

### Sensitivity Analyses

Results from the pattern‐mixture models (see Table 4), attenuated the association between uric acid and MDS‐UPDRS III slope (*P* = 0.10 and q = 0.24). It also slightly attenuated the associations between ApoA1, CRP, and vitamin D and the MDS‐UPDRS II intercept; however, they all remained statistically significant (*P* < 0.05 and q < 0.05). The association between uric acid and the MDS‐UPDRS I intercept was attenuated to the null and no longer significant (*P* = 0.06 and q = 0.11). The association between ApoA1 and the MDS‐UPDRS I intercept was no longer significant after adjusting for multiple comparisons (q = 0.08).

**Table 4:** Pattern mixture model (adjusted for withdrawal) longitudinal follow‐up associations (per standard deviation change in transformed biomarker)

	Adjusted associations			
MDS‐UPDRS III	Intercept	Slope (per year)	Intercept q value	Slope q value
ApoA1	−0.93 (−2.02 to 0.16); 0.10	0.04 (−0.30 to 0.38); 0.82	0.36	0.90
CRP	−0.40 (−1.40 to 0.60); 0.43	0.26 (−0.07 to 0.58); 0.12	0.43	0.24
Uric acid^a^	−0.70 (−1.82 to 0.41); 0.22	0.29 (−0.05 to 0.64); 0.10	0.36	0.24
Vitamin D	−0.65 (−1.79 to 0.50); 0.27	−0.02 (−0.38 to 0.33); 0.90	0.36	0.90
MoCA	Adjusted Associations			
	Intercept	Slope (per year)	Intercept q value	Slope q value
ApoA1	0.29 (−0.04 to 0.62); 0.08	−0.02 (−0.11 to 0.07); 0.64	0.23	0.72
CRP	−0.17 (−0.46 to 0.12); 0.24	−0.03 (−0.12 to 0.06); 0.57	0.28	0.72
Uric acid^a^	0.25 (−0.06 to 0.55); 0.12	−0.05 (−0.14 to 0.05); 0.32	0.23	0.72
Vitamin D	0.16 (−0.12 to 0.44); 0.28	0.02 (−0.07 to 0.10); 0.72	0.28	0.72
MDS‐UPDRS II	Adjusted associations			
	Intercept	Slope (per year)	Intercept q value	Slope q value
**ApoA1**	−0.73 (−1.33 to −0.13); 0.02	−0.16 (−0.35 to 0.02); 0.08	0.02	0.32
CRP	0.76 (0.18 to 1.34); 0.01	0.11 (−0.05 to 0.27); 0.17	0.02	0.33
Uric acid^a^	0.07 (−0.52 to 0.66); 0.82	0.06 (−0.11 to 0.24); 0.48	0.82	0.48
Vitamin D	−0.74 (−1.31 to −0.17); 0.01	−0.06 (−0.23 to 0.11); 0.48	0.02	0.48
MDS‐UPDRS I	Adjusted associations			
	Intercept	Slope (per year)	Intercept q value	Slope q value
ApoA1	−0.65 (−1.20 to −0.11); 0.02	−0.10 (−0.26 to 0.07); 0.25	0.08	0.49
CRP	0.40 (−0.10 to 0.90); 0.12	0.08 (−0.06 to 0.23); 0.24	0.12	0.49
Uric acid^a^	0.50 (−0.01 to 1.00); 0.06	−0.03 (−0.16 to 0.10); 0.63	0.11	0.79
Vitamin D	−0.44 (−0.99 to 0.11); 0.12	0.02 (−0.11 to 0.14); 0.79	0.12	0.79

MDS‐UPDRS, Movement Disorder Society Unified Parkinson's Disease Rating Scale; MoCA, Montreal Cognitive Assessment; ApoA1, apolipoprotein A1; CRP, C‐reactive protein.

Data (except where stated) are estimate (95% confidence interval); *P* value. Models are adjusted for age at diagnosis and sex.

a Uric acid was standardized by sex but the adjusted associations are still adjusted with a sex term in the model because the interaction between sex and withdrawal has residual confounding with the standardized uric acid.

The results from our sensitivity analysis in which we imputed data if 80% or more of the questions were answered (see web Table 3 for adjusted results and web Table 4 for crude results) were similar to our main analysis, although one could argue the association between CRP and the MDS‐UPDRS III slope was strengthened slightly. Also, the results from our sensitivity analysis adjusting MDS‐UPDRS II and III for LEDD (see web Table 5 for adjusted results and web Table 6 for crude results) were similar to the main analysis.

**Table 5:** Biomarkers versus age at onset and age at diagnosis adjusted for sex and disease duration

Blood biomarker	Direction	*P* age at onset	*P* age at diagnosis	q Value age at onset	q Value age at diagnosis
ApoA1	‐	0.19	0.21	0.26	0.28
CRP	+	0.05	0.045	0.10	0.09
Uric acid	+	0.02	0.03	0.09	0.09
Vitamin D	+	0.58	0.65	0.58	0.65

ApoA1, apolipoprotein A1; CRP, C‐reactive protein.

#### Biomarker versus age at onset and diagnosis

Raised uric acid was associated with higher age at onset (*P* = 0.02) and higher age at diagnosis (*P* = 0.03), see Table 5, but were not significant after adjustment for multiple comparisons (q = 0.09 for both age at onset and diagnosis). There were borderline associations between raised CRP, higher age at onset (*P* = 0.05), and higher age at diagnosis (*P* = 0.045); however, these were not significant after adjustment for multiple comparisons (q = 0.10 and q = 0.09, respectively).

## Discussion

Baseline clinical subtyping approaches identified a proinflammatory biomarker profile (reduced apolipoprotein A1 and raised CRP) significantly associated with the severe motor and nonmotor disease phenotype, lending biological validity to this approach. Although after adjustment for covariables and accounting for withdrawal, none of our 4 blood biomarkers were associated with prognosis (slope), but 3 of the biomarkers (ApoA1, CRP, and vitamin D) were associated with baseline disease severity (intercept) in the case of MDS‐UPDRS II.

In this study, we found strong differences between our biomarkers and data‐derived PD subtypes derived from a wealth of baseline phenotypic data encompassing motor, nonmotor, and cognitive measures using a previously published unbiased approach.^32^, ^33^ These subtypes were developed and validated in 1601 and 944 recently diagnosed idiopathic PD patients from the Tracking Parkinson's and Discovery cohorts, respectively, followed up over a median of 3 years. The identified 4 clusters were: (1) fast motor progression with symmetrical motor disease, poor olfaction, cognition, and postural hypotension; (2) mild motor and nonmotor disease with intermediate motor progression; (3) severe motor disease, poor psychological well‐being, and poor sleep with an intermediate motor progression; and (4) slow motor progression with tremor‐dominant unilateral disease. The third cluster in this study comprised 22% of the combined baseline PD cohorts. These clusters were associated with response to levodopa in a levodopa challenge and also with motor progression rates.

In the current study, patients assigned to cluster 3 (severe disease group) had significantly reduced ApoA1 and higher CRP levels compared with patients in other clusters, suggestive of a proinflammatory state. This provides support to a growing consensus that both central brain and peripheral blood immune activation are relevant to PD and neurodegenerations.^28^, ^39^, ^40^, ^41^ We also found associations between reduced ApoA1 and higher CRP levels with worse baseline MDS‐UPDRS II. Reduced ApoA1 and raised CRP have also been described in a number of systemic conditions including colorectal carcinoma, in which serum ApoA1 level showed a strong negative correlation with systemic markers of inflammation including serum CRP and serum interleukin‐8 levels. Furthermore, reduced ApoA1 has predicted poor survival in cancer^40^ and associations with earlier age at onset and greater motor severity in PD.^42^ We failed to replicate these associations between ApoA1 with age at onset and cross‐sectional associations with UPDRS‐III. The PPMI study also failed to show an association with ApoA1 and motor decline, albeit over a very short period of only 12 months, which may be too short to detect differences.^42^ Another incident PD cohort showed similar proinflammatory response markers in the serum of incident PD subjects.^31^ They also found that higher CRP predicted faster rates of change in UPDRS‐III over a 3‐year period, which we failed to replicate in our own cohort.

One interesting question is why our clusters associated with motor progression rates and also with ApoA1 and CRP, but ApoA1 and CRP did not associate with motor progression rates. This is because of our third cluster, which drove the associations with ApoA1 and CRP, having intermediate progression rates, whereas cluster 1, with the fastest rate of disease progression, did not have any association with the biomarkers.

Immune activation is described in many neurodegenerative disorders including PD in the brain and peripheral blood, leading to the hypothesis that there are common mechanisms in these disorders through which the immune system mediates disease initiation and/or progression. This is of considerable interest given that the immune system is a highly tractable therapeutic target and that therapeutic approaches targeting protein aggregation have not yet shown efficacy in clinical trials. The critical question of whether immune changes are a primary driver in their pathogenesis is difficult to address because significant neurodegeneration has already occurred when PD patients first become symptomatic. Future serum analysis work will focus on our prodromal subjects who have polysomnographically diagnosed rapid eye movement sleep behavior disorder (RBD) and share both nonmotor and motor features in common with early PD.

This study adds considerable evidence to the literature on each of these analytes. Conflicting evidence for the use of vitamin D and uric acid in multiple studies has led to uncertainty over their utility to predict outcomes in early PD, despite the precious resources used to investigate them. Studies investigating ApoA1 and CRP have been promising, but the same results failed to be replicated in this large prospective cohort, and we found no differences with progression rates in 4 domains. Therefore, this study should guide future research toward other prognostic candidates, at least in the short‐term follow‐up of 3 years.

The association we found between uric acid and MDS‐UPDRS III is interesting but also raises challenges in its interpretation. The mounting evidence is that in Parkinson's disease higher uric acid levels are protective or better.^22^, ^23^ Our model seems to suggest that there is a weak association between higher uric acid and lower MDS‐UPDRS III intercept (*P* = 0.08), but a slightly stronger association between higher uric acid level and higher MDS‐UPDRS III slope (*P* = 0.03). Furthermore, looking at the associations between uric acid and MDS‐UPDRS I intercept, these occur in the direction one would expect if higher uric acid level predicted worse nonmotor function. Accounting for withdrawal and multiple comparisons further attenuated any association with progression and intercept. It is not clear why our findings of a weak association with uric acid level was not consistent with observational studies, suggesting an etiological protective effect.^21^, ^43^ Phase 2 intervention studies have been undertaken showing therapeutic intervention to increase uric acid is safe^44^; however, a large phase 3 trial (SURE‐PD3 NCT02642393) that was expected to end in 2020 was terminated early due to futility. This provides more evidence that higher uric acid is not causally associated with better prognosis. It should be noted that both observational studies^22^, ^23^ and the Mendelian randomization study (carried out on the same 2 populations) linking uric acid level were based on individuals who had not been exposed to dopaminergic therapy, with the main outcome progression to disability requiring dopaminergic therapy. By excluding those who required dopaminergic therapy close to diagnosis, these studies might be based on individuals whose motor symptoms were relatively mild, whereas in our study only 11.9% remained untreated at recruitment.

It should be noted that our results provide evidence of a cross‐sectional association between serum levels and PD disease markers. As we have not individually measured each serum biomarker longitudinally, the directionality of each association remains unclear, and therefore could merely reflect the secondary effects of disease severity on the serum marker. As these serum levels are associated with baseline severity and not disease progression, they could be a state marker of the disease rather than a trait marker of a more malignant disease process. The proinflammatory response strongly associated with cluster 3 and baseline UPDRS‐II could reflect a different pathophysiology driven by neuroinflammation. Previous work associating CRP with coronary heart disease severity at a cross‐sectional level was not subsequently replicated by larger Mendelian randomization studies,^37^ suggesting that the results were the effect of reverse causation or a secondary effect of disease severity.

It is intriguing to speculate that because MDS‐UPDRS I and II are patient completed, there may be some between‐individual elements like lifestyle factors that have confounded our associations between the biomarkers and the MDS‐UPDRS I and II intercepts. We could argue about some *P* values being on the border of significance and further follow‐up or a larger sample with increased power might find similar associations but with smaller confidence intervals. However, when examining the confidence intervals, the effect sizes all tended to be on the small side. For instance, our significant results between the UPDRS II intercept and 3 of the biomarkers were at most (see 95% confidence interval of main analysis) a difference of 1.5 UPDRS II points for a change of 1 standard deviation in the biomarker and at least a 0.2 UPDRS II points for a change of 1 standard deviation.

### Strengths, Limitations, and Conclusions

The Discovery cohort is one of the largest PD incidence cohorts worldwide and currently has more than 3 years of follow‐up on average. We have a wealth of phenotypic information that went into our data‐derived PD subtypes that have been developed and validated in 2 incidence cohorts using > 2500 patients.

With more follow‐up data, we will be able to consider interesting questions about nonlinearity and complex measurement error. Our current follow‐up period may be too short to detect different progression rates. There are some problems with using corrections to *P* values,^45^ and although the authors focused on statistical significance in the Results section, they were also aware that there are pitfalls to this that have been highlighted before^46^, ^47^ However, any adjustment we could make to our *P* values for multiple testing would further justify our conclusion that the biomarkers we tested are not related to prognosis in any of the 4 domains we studied. For those reaching each visit, the amount of missing data was very small, and our adjustments for individuals whose entire questionnaires were not completed showed similar associations between the biomarkers and the outcomes. We also used robust methods (pattern‐mixture models) to take into account individuals who had withdrawn from the study, which could bias our estimates, as individuals with more severe disease were more likely to withdraw. We also attempted to exclude individuals who may have been misclassified as PD but turned out to have other parkinsonian disorders. Our associations with vitamin D might not be generalizable to people at different latitudes.

Our models would give a better picture of progression had we measured the blood biomarkers at each visit rather than just at baseline. However, the current study design was sufficient to interrogate the role of baseline serum in influencing future progression. Further studies should focus on longitudinal serum measurements and on earlier disease cohorts including patients with prodromal RBD sleep disorder, and carriers of monogenic mutations strongly implicated in PD pathogenesis, such as the glucocerebrosidase (*GBA*) gene. This will address the critical question of whether immune changes are the primary driver in PD pathogenesis or a secondary effect of worsening disease burden.

In future work we plan to examine the genetics of our clusters. This includes whether certain clusters are enriched with *GBA* or LRRK2 carriers, whether clusters differ in their genetic risk of developing PD, and carrying out a genome‐wide association study of belonging to a cluster.

Last, given that therapeutic approaches targeting protein aggregation in PD have not yet shown efficacy in clinical trials, the immune system remains a highly tractable therapeutic target. Our data suggest that a simple, low‐cost serum‐based approach could both stratify PD patients most likely to benefit from immunomodulatory approaches and monitor future treatment response, both of which are critically needed if we are to really deliver neuroprotective interventions for PD.^48^

## Author contributions

Michael Lawton — analysis and interpretation of the data, writing of the manuscript.

Fahd Baig — study concept and design, acquisition of data, writing and revision of the manuscript.

Greg Toulson — acquisition of data, revision of the manuscript.

Alireza Morovat — acquisition of data, revision of the manuscript.

Samuel G. Evetts — acquisition of data, revision of the manuscript.

Yoav Ben‐Shlomo — study concept and design, analysis and interpretation of the data, revision of the manuscript.

Michele T.M. Hu — study concept and design, acquisition of data, revision of the manuscript.

## Ethics approval

Local NHS ethics committee.

## Relevant conflicts of interest/financial disclosures

The authors have no conflict of interest to report. Michael Lawton is employed on Parkinson's UK grant. Fahd Baig is employed by Oxford Parkinson's Disease Centre, North Bristol NHS Trust and Nottingham University Trust. Greg Toulson is employed by Oxford University Hospital NHS Foundation Trust and Birmingham Children's Hospital NHS Trust. Alireza Morovat is employed by Oxford University Hospital NHS Foundation Trust. Samuel G. Evetts is employed on a Parkinson's UK grant. Yoav Ben‐Shlomo receives grants from Parkinson's UK, NIHR, MRC, Gatsby Foundation, Kidney Research UK, and Brain Tumour Charity. Michele T. Hu receives grants from Parkinson's UK, Oxford NIHR Biomedical Research Centre, and MJFF and is an adviser to the Roche Prodromal Advisory and Biogen Digital advisory boards.

## Funding agencies

This study was funded by the Monument Trust Discovery Award from Parkinson**'**s UK and supported by the National Institute for Health Research (NIHR) Oxford Biomedical Research Centre based at Oxford University Hospitals NHS Trust, and University of Oxford and the Dementias and Neurodegenerative Diseases Research Network (DeNDRoN). The views expressed are those of the author(s) and not necessarily those of the NHS, the NIHR, or the Department of Health. The biochemical analysis was funded by a Parkinson's UK innovation grant (INN‐14A‐37).

## Supporting information


**Web table 1**. Amount of longitudinal data available on all 624 PD patients. Note that the number of visits and withdrawals is different for the MDS‐UPDRS II and MDS‐UPDRS I as it is patient completed and still available for those on telephone only and not clinic visits

**Web Table 2**. Crude longitudinal follow‐up associations (per sd change in transformed biomarker) that relate to table 3 from the main article. Data (except where stated) is estimate (95% confidence interval); p‐value.

**Web Table 3**. Sensitivity analysis where we imputed missing data in the case where at least 80% of the questionnaire were completed. Longitudinal follow‐up associations (per sd change in transformed biomarker). Data (except where stated) is estimate (95% confidence interval); p‐value. Models are adjusted for age at diagnosis and gender.

**Web Table 4**. Crude longitudinal follow‐up associations (per sd change in transformed biomarker) that relate to web table 3. Sensitivity analysis where we imputed missing data in the case where at least 80% of the questionnaire were completed. Data (except where stated) is estimate (95% confidence interval); p‐value.

**Web Table 5**. Sensitivity analysis where we adjusted MDS‐UPDRS II and III for levodopa equivalent daily dose. Longitudinal follow‐up associations (per sd change in transformed biomarker). Data (except where stated) is estimate (95% confidence interval); p‐value. Models are adjusted for age at diagnosis and gender.

**Web Table 6**. Crude longitudinal follow‐up associations (per sd change in transformed biomarker) that relate to web table 5. Sensitivity analysis where we adjusted MDS‐UPDRS II and III for levodopa equivalent daily dose. Data (except where stated) is estimate (95% confidence interval); p‐value.

**Web Figure 1**. Graphical representation of longitudinal associations with intercept and slope. Figure 1.a is top left representing an association with the intercept, figure 1.b is top right representing an association with the slope only and 1.c is bottom left representing an association with both the intercept and slope.

**Web figure 2**. Flow chart to show entry into this study

**Appendix S1:** Supporting Information
