# Supplementary material for: Blood biomarkers with Parkinson's disease clusters and prognosis: The oxford discovery cohort
Source: Mov Disord. 2019 Nov 6;35(2):279–87. doi: 10.1002/mds.27888 (PMC7028059; doi:10.1002/mds.27888)
Supplement: Supplementary file 1 — Web table 1. Amount of longitudinal data available on all 624 PD patients. Note that the number of visits and withdrawals is different for the MDS‐UPDRS II and MDS‐UPDRS I as it is patient completed and still available for those on telephone only and not clinic visits [file MDS-35-279-s001.docx]

**Web table 1.** Amount of longitudinal data available on all 624 PD patients. Note that the number of visits and withdrawals is different for the MDS-UPDRS II and MDS-UPDRS I as it is patient completed and still available for those on telephone only and not clinic visits

| **Visit Number: proportion with data** | **Cumulative number of withdrawals** | **MDS-UPDRS III data amount** | **MDS-UPDRS III data amount (80% answered)** | **MoCA data amount** | **MoCA data amount (80% answered)** |
| --- | --- | --- | --- | --- | --- |
| 1: 624 (100%) |  | 624/624 (100%) | 624/624 (100%) | 624/624 (100%) | 624/624 (100%) |
| 2: 527 (84.5%) | 74: 11.9% | 495/527 (93.9%) | 519/527 (98.5%) | 514/527 (97.5%) | 522/527 (99.1%) |
| 3: 394 (63.1%) | 163: 26.1% | 383/394 (97.2%) | 390/394 (99.0%) | 383/394 (97.2%) | 387/394 (98.2%) |
| 4: 191 (30.6%) | 218: 34.9% | 175/191 (91.6%) | 186/191 (97.4%) | 182/191 (95.3%) | 189/191 (99.0%) |
| 5: 67 (10.7%) | 227: 36.4% | 59/67 (88.1%) | 66/67 (98.5%) | 66/67 (98.5%) | 67/67 (100.0%) |
| **Visit Number: proportion with data including telephone visits** | **Cumulative number of withdrawals** | **MDS-UPDRS II data amount** | **MDS-UPDRS II data amount (80% answered)** | **MDS-UPDRS I data amount** | **MDS-UPDRS I data amount (80% answered)** |
| 1: 624 (100%) |  | 615/624 (98.6%) | 619/624 (99.2%) | 615/624 (98.6%) | 617/624 (98.9%) |
| 2: 533 (85.4%) | 63: 10.1% | 517/533 (97.0%) | 520/533 (97.6%) | 516/533 (96.8%) | 520/533 (97.6%) |
| 3: 423 (67.8%) | 131: 21.0% | 405/423 (95.7%) | 409/423 (96.7%) | 400/423 (94.7%) | 404/423 (95.5%) |
| 4: 242 (38.8%) | 175: 28.0% | 226/242 (93.4%) | 226/242 (93.4%) | 224/242 (92.6%) | 225/242 (93.0%) |
| 5: 96 (15.4%) | 196: 31.4% | 95/96 (99.0%) | 96/96 (100.0%) | 95/96 (99.0%) | 95/96 (99.0%) |

MDS-UPDRS = Movement Disorder Society Unified Parkinson’s Disease Rating Scale, MoCA = Montreal Cognitive Assessment
