# Supplementary material for: Blood biomarkers with Parkinson's disease clusters and prognosis: The oxford discovery cohort
Source: Mov Disord. 2019 Nov 6;35(2):279–87. doi: 10.1002/mds.27888 (PMC7028059; doi:10.1002/mds.27888)
Supplement: Supplementary file 2 — Web Table 2. Crude longitudinal follow‐up associations (per sd change in transformed biomarker) that relate to table 3 from the main article. Data (except where stated) is estimate (95% confidence interval); p‐value. [file MDS-35-279-s002.docx]

**Web Table 2.** Crude longitudinal follow-up associations (per sd change in transformed biomarker) that relate to table 3 from the main article.

| **MDS-UPDRS III** | **CRUDE ASSOCIATIONS** | |  |  |
| --- | --- | --- | --- | --- |
|  | **Intercept** | **Slope (per year)** | **Intercept q-value** | **Slope q-value** |
| **ApoA1** | -0.77 (-1.83 to 0.29); 0.15 | -0.26 (-0.57 to 0.05); 0.10 | 0.23 | 0.14 |
| **CRP** | -0.16 (-1.23 to 0.90); 0.76 | 0.29 (-0.03 to 0.61); 0.07 | 0.76 | 0.14 |
| **Uric acid** | -0.78 (-1.86 to 0.31); 0.16 | 0.42 (0.09 to 0.74); 0.01 | 0.23 | 0.047 |
| **Vitamin D** | -0.75 (-1.82 to 0.33); 0.18 | 0.04 (-0.27 to 0.36); 0.78 | 0.23 | 0.78 |
|  |  |  |  |  |
| **MoCA** | **CRUDE ASSOCIATIONS** | |  |  |
|  | **Intercept** | **Slope (per year)** | **Intercept q-value** | **Slope q-value** |
| **ApoA1** | 0.40 (0.11 to 0.69); 0.007 | 0.02 (-0.05 to 0.09); 0.56 | 0.03 | 0.58 |
| **CRP** | -0.27 (-0.56 to 0.02); 0.07 | -0.03 (-0.11 to 0.04); 0.39 | 0.14 | 0.58 |
| **Uric acid** | 0.12 (-0.18 to 0.43); 0.42 | -0.04 (-0.11 to 0.04); 0.31 | 0.42 | 0.58 |
| **Vitamin D** | 0.12 (-0.17 to 0.42); 0.41 | 0.02 (-0.05 to 0.09); 0.58 | 0.42 | 0.58 |
|  |  |  |  |  |
| **MDS-UPDRS II** | **CRUDE ASSOCIATIONS** | |  |  |
|  | **Intercept** | **Slope (per year)** | **Intercept q-value** | **Slope q-value** |
| **ApoA1** | -0.82 (-1.34 to -0.31); 0.002 | -0.14 (-0.27 to -0.01); 0.04 | 0.004 | 0.13 |
| **CRP** | 0.83 (0.30 to 1.35); 0.002 | 0.13 (-0.01 to 0.26); 0.06 | 0.004 | 0.13 |
| **Uric acid** | 0.07 (-0.46 to 0.60); 0.80 | 0.07 (-0.07 to 0.20); 0.32 | 0.80 | 0.32 |
| **Vitamin D** | -0.74 (-1.27 to -0.21); 0.006 | -0.08 (-0.21 to 0.05); 0.25 | 0.008 | 0.32 |
|  |  |  |  |  |
| **MDS-UPDRS I** | **CRUDE ASSOCIATIONS** | |  |  |
|  | **Intercept** | **Slope (per year)** | **Intercept q-value** | **Slope q-value** |
| **ApoA1** | -0.46 (-0.91 to -0.02); 0.04 | -0.05 (-0.15 to 0.05); 0.29 | 0.06 | 0.48 |
| **CRP** | 0.48 (0.03 to 0.93); 0.04 | 0.07 (-0.04 to 0.17); 0.20 | 0.06 | 0.48 |
| **Uric acid** | 0.56 (0.11 to 1.02); 0.02 | -0.05 (-0.15 to 0.06); 0.36 | 0.06 | 0.48 |
| **Vitamin D** | -0.43 (-0.89 to 0.02); 0.06 | -0.02 (-0.13 to 0.08); 0.66 | 0.06 | 0.66 |

MDS-UPDRS = Movement Disorder Society Unified Parkinson’s Disease Rating Scale, MoCA = Montreal Cognitive Assessment, ApoA1 = Apolipoprotein A1, CRP = C-Reactive Protein

Data (except where stated) is estimate (95% confidence interval); p-value.
