# Supplementary material for: Blood biomarkers with Parkinson's disease clusters and prognosis: The oxford discovery cohort
Source: Mov Disord. 2019 Nov 6;35(2):279–87. doi: 10.1002/mds.27888 (PMC7028059; doi:10.1002/mds.27888)
Supplement: Supplementary file 3 — Web Table 3. Sensitivity analysis where we imputed missing data in the case where at least 80% of the questionnaire were completed. Longitudinal follow‐up associations (per sd change in transformed biomarker). Data (except where stated) is estimate (95% confidence interval); p‐value. Models are adjusted for age at diagnosis and gender. [file MDS-35-279-s003.docx]

**Web Table 3.** Sensitivity analysis where we imputed missing data in the case where at least 80% of the questionnaire were completed. Longitudinal follow-up associations (per sd change in transformed biomarker).

| **MDS-UPDRS III** | **ADJUSTED ASSOCIATIONS** | |  |  |
| --- | --- | --- | --- | --- |
|  | **Intercept** | **Slope (per year)** | **Intercept q-value** | **Slope q-value** |
| **ApoA1** | -0.85 (-1.99 to 0.29); 0.14 | -0.07 (-0.41 to 0.26); 0.67 | 0.19 | 0.89 |
| **CRP** | -0.43 (-1.46 to 0.60); 0.41 | 0.30 (-0.00 to 0.61); 0.053 | 0.41 | 0.11 |
| **Uric acid^1^** | -0.93 (-1.98 to 0.12); 0.08 | 0.33 (0.01 to 0.64); 0.04 | 0.19 | 0.11 |
| **Vitamin D** | -0.80 (-1.85 to 0.25); 0.13 | 0.02 (-0.29 to 0.32); 0.92 | 0.19 | 0.92 |
|  |  |  |  |  |
| **MoCA** | **ADJUSTED ASSOCIATIONS** | |  |  |
|  | **Intercept** | **Slope (per year)** | **Intercept q-value** | **Slope q-value** |
| **ApoA1** | 0.29 (-0.02 to 0.60); 0.07 | 0.00 (-0.08 to 0.08); 0.98 | 0.20 | 0.98 |
| **CRP** | -0.21 (-0.49 to 0.07); 0.15 | -0.03 (-0.10 to 0.04); 0.45 | 0.20 | 0.70 |
| **Uric acid^1^** | 0.22 (-0.07 to 0.50); 0.14 | -0.03 (-0.11 to 0.04); 0.41 | 0.20 | 0.70 |
| **Vitamin D** | 0.16 (-0.12 to 0.45); 0.26 | 0.02 (-0.05 to 0.10); 0.52 | 0.26 | 0.70 |
|  |  |  |  |  |
| **MDS-UPDRS II** | **ADJUSTED ASSOCIATIONS** | |  |  |
|  | **Intercept** | **Slope (per year)** | **Intercept q-value** | **Slope q-value** |
| **ApoA1** | -0.94 (-1.51 to -0.37); 0.001 | -0.06 (-0.20 to 0.09); 0.44 | 0.004 | 0.57 |
| **CRP** | 0.83 (0.30 to 1.35); 0.002 | 0.11 (-0.02 to 0.24); 0.11 | 0.004 | 0.37 |
| **Uric acid^1^** | 0.05 (-0.48 to 0.58); 0.86 | 0.04 (-0.10 to 0.17); 0.57 | 0.86 | 0.57 |
| **Vitamin D** | -0.77 (-1.30 to -0.25); 0.004 | -0.09 (-0.22 to 0.04); 0.19 | 0.005 | 0.37 |
|  |  |  |  |  |
| **MDS-UPDRS I** | **ADJUSTED ASSOCIATIONS** | |  |  |
|  | **Intercept** | **Slope (per year)** | **Intercept q-value** | **Slope q-value** |
| **ApoA1** | -0.82 (-1.31 to -0.34); <0.001 | -0.01 (-0.12 to 0.10); 0.80 | 0.004 | 0.80 |
| **CRP** | 0.48 (0.03 to 0.93); 0.04 | 0.06 (-0.05 to 0.16); 0.29 | 0.047 | 0.62 |
| **Uric acid^1^** | 0.53 (0.07 to 0.98); 0.02 | -0.05 (-0.16 to 0.05); 0.31 | 0.046 | 0.62 |
| **Vitamin D** | -0.41 (-0.87 to 0.04); 0.07 | -0.03 (-0.13 to 0.07); 0.58 | 0.07 | 0.78 |

MDS-UPDRS = Movement Disorder Society Unified Parkinson’s Disease Rating Scale, MoCA = Montreal Cognitive Assessment, ApoA1 = Apolipoprotein A1, CRP = C-Reactive Protein

Data (except where stated) is estimate (95% confidence interval); p-value. Models are adjusted for age at diagnosis and gender.

^1^Uric acid was standardised by gender so the adjusted associations are not adjusted with a gender term in the model
