# Supplementary material for: Blood biomarkers with Parkinson's disease clusters and prognosis: The oxford discovery cohort
Source: Mov Disord. 2019 Nov 6;35(2):279–87. doi: 10.1002/mds.27888 (PMC7028059; doi:10.1002/mds.27888)
Supplement: Supplementary file 5 — Web Table 5. Sensitivity analysis where we adjusted MDS‐UPDRS II and III for levodopa equivalent daily dose. Longitudinal follow‐up associations (per sd change in transformed biomarker). Data (except where stated) is estimate (95% confidence interval); p‐value. Models are adjusted for age at diagnosis and gender. [file MDS-35-279-s005.docx]

**Web Table 5.** Sensitivity analysis where we adjusted MDS-UPDRS II and III for levodopa equivalent daily dose. Longitudinal follow-up associations (per sd change in transformed biomarker).

| **MDS-UPDRS III** | **ADJUSTED ASSOCIATIONS** | |  |  |
| --- | --- | --- | --- | --- |
|  | **Intercept** | **Slope (per year)** | **Intercept q-value** | **Slope q-value** |
| **ApoA1** | -1.09 (-2.26 to 0.07); 0.07 | -0.05 (-0.40 to 0.30); 0.79 | 0.09 | 0.79 |
| **CRP** | -0.11 (-1.16 to 0.94); 0.84 | 0.23 (-0.09 to 0.54); 0.16 | 0.84 | 0.31 |
| **Uric acid^1^** | -1.09 (-2.17 to -0.02); 0.046 | 0.40 (0.07 to 0.72); 0.02 | 0.09 | 0.06 |
| **Vitamin D** | -1.02 (-2.08 to 0.05); 0.06 | 0.05 (-0.26 to 0.36); 0.76 | 0.09 | 0.79 |
|  |  |  |  |  |
| **MDS-UPDRS II** | **ADJUSTED ASSOCIATIONS** | |  |  |
|  | **Intercept** | **Slope (per year)** | **Intercept q-value** | **Slope q-value** |
| **ApoA1** | -1.02 (-1.62 to -0.42); <0.001 | -0.05 (-0.19 to 0.10); 0.53 | 0.003 | 0.71 |
| **CRP** | 0.85 (0.31 to 1.40); 0.002 | 0.10 (-0.03 to 0.24); 0.14 | 0.004 | 0.56 |
| **Uric acid^1^** | 0.06 (-0.49 to 0.62); 0.83 | 0.02 (-0.12 to 0.16); 0.81 | 0.83 | 0.81 |
| **Vitamin D** | -0.84 (-1.39 to -0.29); 0.003 | -0.06 (-0.20 to 0.07); 0.35 | 0.004 | 0.70 |

MDS-UPDRS = Movement Disorder Society Unified Parkinson’s Disease Rating Scale, MoCA = Montreal Cognitive Assessment, ApoA1 = Apolipoprotein A1, CRP = C-Reactive Protein

Data (except where stated) is estimate (95% confidence interval); p-value. Models are adjusted for age at diagnosis and gender.

^1^Uric acid was standardised by gender so the adjusted associations are not adjusted with a gender term in the model
