# Supplementary material for: Blood biomarkers with Parkinson's disease clusters and prognosis: The oxford discovery cohort
Source: Mov Disord. 2019 Nov 6;35(2):279–87. doi: 10.1002/mds.27888 (PMC7028059; doi:10.1002/mds.27888)
Supplement: Supplementary file 6 — Web Table 6. Crude longitudinal follow‐up associations (per sd change in transformed biomarker) that relate to web table 5. Sensitivity analysis where we adjusted MDS‐UPDRS II and III for levodopa equivalent daily dose. Data (except where stated) is estimate (95% confidence interval); p‐value. [file MDS-35-279-s006.docx]

**Web Table 6.** Crude longitudinal follow-up associations (per sd change in transformed biomarker) that relate to web table 5. Sensitivity analysis where we adjusted MDS-UPDRS II and III for levodopa equivalent daily dose.

| **MDS-UPDRS III** | **CRUDE ASSOCIATIONS** | |  |  |
| --- | --- | --- | --- | --- |
|  | **Intercept** | **Slope (per year)** | **Intercept q-value** | **Slope q-value** |
| **ApoA1** | -0.90 (-1.99 to 0.18); 0.10 | -0.28 (-0.60 to 0.04); 0.09 | 0.15 | 0.17 |
| **CRP** | 0.11 (-0.97 to 1.19); 0.85 | 0.24 (-0.08 to 0.56); 0.14 | 0.85 | 0.18 |
| **Uric acid** | -0.89 (-1.99 to 0.22); 0.12 | 0.45 (0.12 to 0.77); 0.007 | 0.15 | 0.03 |
| **Vitamin D** | -0.93 (-2.03 to 0.16); 0.10 | 0.07 (-0.25 to 0.39); 0.66 | 0.15 | 0.66 |
|  |  |  |  |  |
| **MDS-UPDRS II** | **CRUDE ASSOCIATIONS** | |  |  |
|  | **Intercept** | **Slope (per year)** | **Intercept q-value** | **Slope q-value** |
| **ApoA1** | -0.86 (-1.40 to -0.31); 0.002 | -0.14 (-0.28 to -0.01); 0.04 | 0.004 | 0.14 |
| **CRP** | 0.88 (0.33 to 1.42); 0.002 | 0.12 (-0.02 to 0.26); 0.10 | 0.004 | 0.19 |
| **Uric acid** | 0.10 (-0.46 to 0.65); 0.73 | 0.04 (-0.10 to 0.18); 0.58 | 0.73 | 0.58 |
| **Vitamin D** | -0.83 (-1.39 to -0.28); 0.003 | -0.05 (-0.19 to 0.09); 0.52 | 0.004 | 0.58 |

MDS-UPDRS = Movement Disorder Society Unified Parkinson’s Disease Rating Scale, MoCA = Montreal Cognitive Assessment, ApoA1 = Apolipoprotein A1, CRP = C-Reactive Protein

Data (except where stated) is estimate (95% confidence interval); p-value.
