# Supplementary figures and images for: Blood biomarkers with Parkinson's disease clusters and prognosis: The oxford discovery cohort
Source: Mov Disord. 2019 Nov 6;35(2):279–87. doi: 10.1002/mds.27888 (PMC7028059; doi:10.1002/mds.27888)

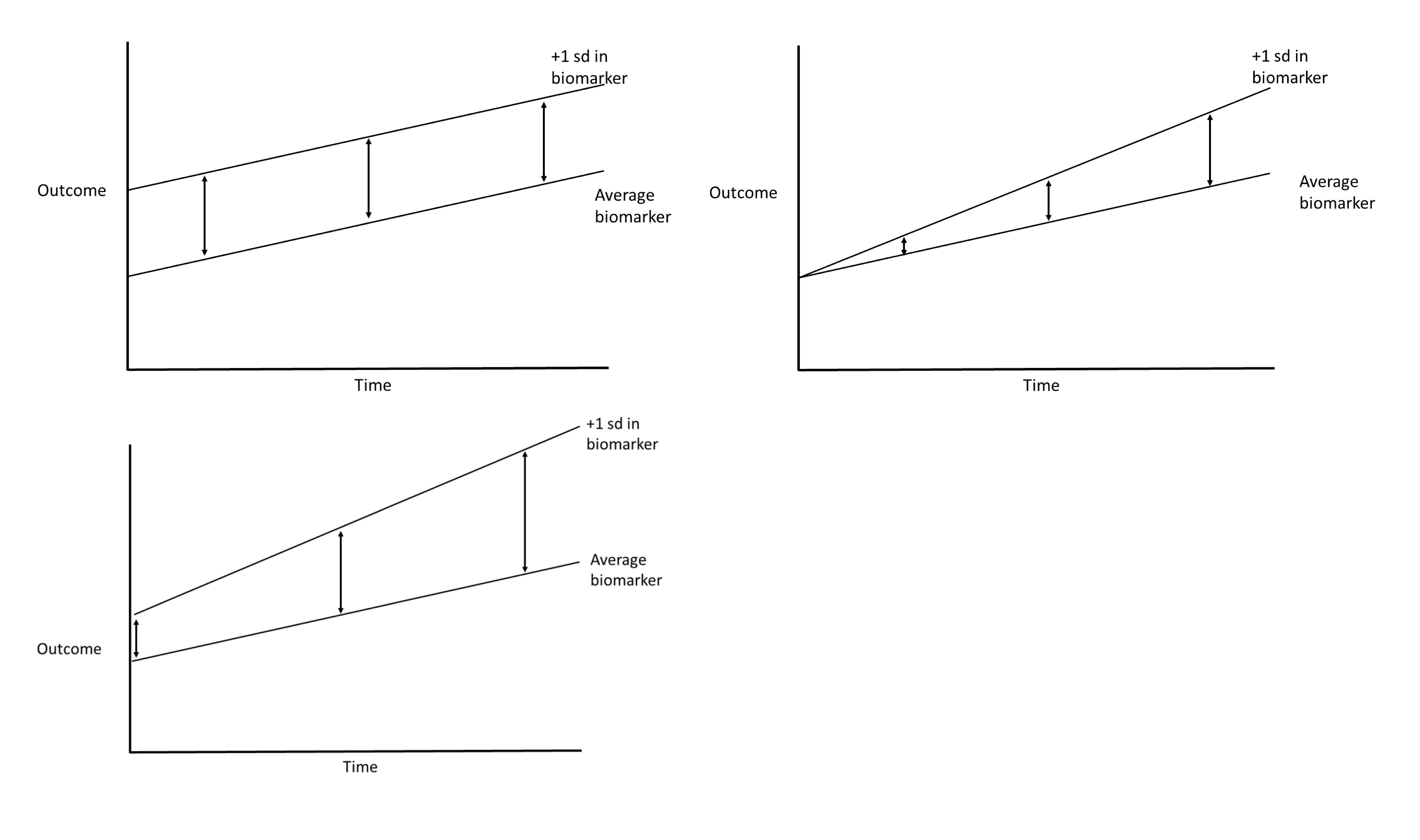

Supplement: Supplementary file 7 — Web Figure 1. Graphical representation of longitudinal associations with intercept and slope. Figure 1.a is top left representing an association with the intercept, figure 1.b is top right representing an association with the slope only and 1.c is bottom left representing an association with both the intercept and slope. [file MDS-35-279-s007.tiff]

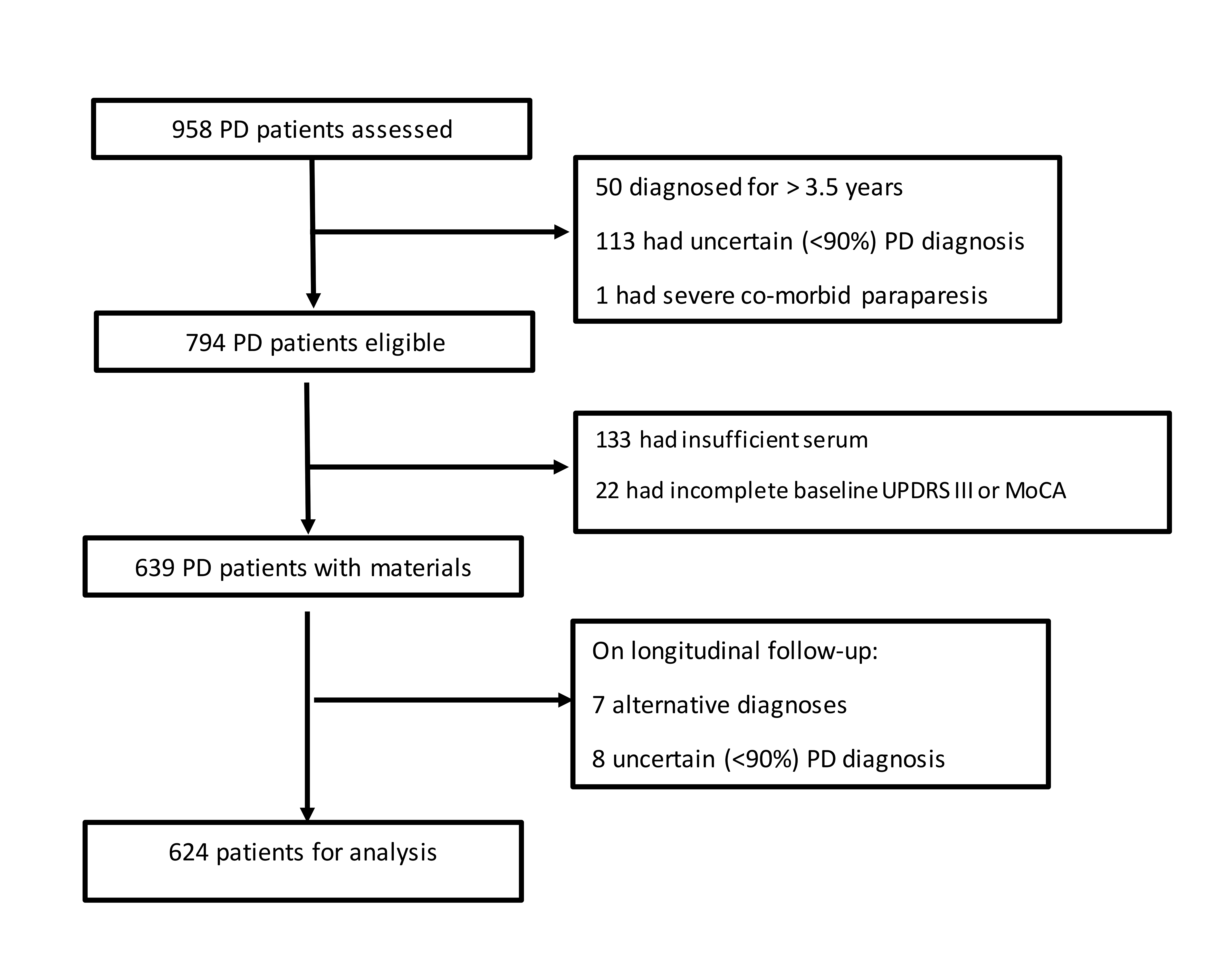

Supplement: Supplementary file 8 — Web figure 2. Flow chart to show entry into this study [file MDS-35-279-s008.tiff]
