# Supplementary material for: Blood biomarkers with Parkinson's disease clusters and prognosis: The oxford discovery cohort
Source: Mov Disord. 2019 Nov 6;35(2):279–87. doi: 10.1002/mds.27888 (PMC7028059; doi:10.1002/mds.27888)
Supplement: Supplementary file 9 — Appendix S1: Supporting Information [file MDS-35-279-s009.docx]

**Web appendix - Supplementary Online Content**

**Blood biomarkers with Parkinson’s disease clusters and prognosis: the Oxford Discovery cohort**

**MATERIALS AND METHODS**

***Measuring serum biomarkers* *- C-reactive protein and Apolipoprotein-A1***

Serum CRP was measured using an immunoturbidimetric assay (Abbott diagnostics, Illinois USA) using a routine automated general chemistry analyser, the Abbot Architect C16000. The methodology used was as recommended by the manufacturer’s instructions (CRP Abbott Architect system kit inset Ref8G65-21 30-4143/R1; ApoA1 Ref 9D92-21 3-4624/R02) and used reagents provided by the manufacturer. An optical sample blank measurement is then performed by the analyser prior to the addition of anti-human CRP. This excess of antibody with sample substrate present causes an increase in sample turbidity due to the formation of insoluble immune complexes. The degree of immunocomplex formation is proportional to the concentration of sample CRP, measured via a reduction in light transmission.

Calibration of the assay was performed in line with the manufacturer’s instructions. Assay performance was assessed using two levels of serum matrix matched quality control material (Technopath, Ballina, Ireland). All quality control values for CRP were within 1 standard deviation from the laboratory running mean and deemed acceptable prior to samples being run. Nine individuals were assigned a value of zero because their levels were below the lower limit of quantitation. The next smallest value was 0.2 mg/L. We gave all nine individuals a value of 0.1 mg/L for analysis since this allowed us to use a log transformation which looked to give the most normal looking distribution.

Assay performance for ApoA1 was assessed using two levels of serum matrix matched quality control material obtained from Technopath. While this assay is intended for diagnostic use, it is not currently deployed as a routine hospital laboratory test. As such, a limited verification of the ApoA1 assay was performed to ascertain if the assay performance was similar to that reported by the manufacturer. Due to limited reagent availability, this was limited to gauging assay linearity, inter-batch precision and interference due to haemolysis. The ELISA used to measure ApoA1 is reliable for relative quantitation, rather than absolute quantitation, permitting the comparison of sample values within a run, but allowing less confidence between runs.

***Measuring serum biomarkers* *- Uric Acid***

Serum uric acid was measured using a two-part enzymatic assay (Abbott diagnostics, Illinois USA) using a routine automated general chemistry analyser, the Abbot Architect C16000. The methodology used was as recommended by the manufacturer’s instructions (Abbott Architect system kit inset Ref 3P39 304647/R02) and uses reagents provided by the manufacturer. The assay utilises a two-part reaction whereby uric acid is first oxidised, producing hydrogen peroxide, which then converts the reagent into quinoneimine dye, which can then be measured as a change in light absorbance at 604nm.

Calibration of the assay was performed using a two-point calibration in line with the manufacturer’s instructions. Assay performance was assessed using two levels of serum matrix matched quality control material (Technopath, Ballina, Ireland). All quality control values were within 1 standard deviation from the laboratory running mean and deemed acceptable prior to samples being run.

***Measuring serum biomarkers* *- Vitamin D***

Serum 25(OH)-VitD was measured using a one-step immunoassay (Abbott diagnostics, Illinois USA) using a routine automated immunochemistry analyser, the Abbott Architect i2000. The methodology used was as recommended by the manufacturer’s instructions (Abbott Architect system kit inset Ref 3L52 49-8941/R02) and utilised the reagents provided by the manufacturer.

Calibration of the assay was performed in line with the manufacturer’s instructions and assay performance was assessed using three levels of serum matrix matched quality control material (Technopath, Ballina, Ireland). All quality control values were within 2 standard deviation from the laboratory running mean and deemed acceptable prior to samples being run.

***Statistical analysis - interpreting associations***

Web figure 1 gives a graphical representation of what we mean by associations with intercept and slope. In Web figure 1.a this shows an association with the intercept only so the difference remains constant over time. In 1.b there is an association with the slope only so the difference changes over time but there is no difference at a time of zero. In 1.c there is an association with both intercept and slope so the difference changes over time and there is also a difference at a time of zero

***Statistical analysis - sensitivity analysis adjusting for LEDD***

It has been shown previously ^1^ that simply adding treatment as a covariate in a regression model is not appropriate where treatment has a direct effect on the outcome. Instead a sensible constant should be added to the outcome so that it could be interpreted as what the outcome would be if the patients remained untreated. We used data from the ELLDOPA study ^2^ (private communications) to quantify how effective different doses of levodopa (150mg, 300mg and 600mg) would be in reducing the UPDRS part II and III scores. We fit a regression model to this data without a constant term (so that a dose of zero would pass through the origin) giving us an equation to adjust UPDRS II and III scores for any levodopa equivalent daily dose. Another problem was that the data from the ELLDOPA study was from the UPDRS and not the MDS-UPDRS ^3^. We used data from a conversion study ^4^ to adjust these equations for MDS-UPDRS differences instead of UPDRS differences. Since the conversions are dependent on Hoehn and Yahr stage we had to ignore the MDS-UPDRS II completed via telephone because the Hoehn and Yahr stage was not collected during telephone follow-up. It was clear that the data was decelerating with increasing dose so we tried fitting models with a square root term. Models with a square term were not plausible as they had a turning point with increasing LEDD past a point being associated with lower adjustment. Our clinical opinion was that a model with a square root only was preferable to a square root with linear term although both had an R-squared of >0.99.

**References**

1. Tobin MD, Sheehan NA, Scurrah KJ, et al. Adjusting for treatment effects in studies of quantitative traits: antihypertensive therapy and systolic blood pressure. *Stat Med* 2005;**24**(19):2911-35.

2. Fahn S, Oakes D, Shoulson I, et al. Levodopa and the progression of Parkinson's disease. *N Engl J Med* 2004;**351**(24):2498-508.

3. Goetz CG, Tilley BC, Shaftman SR, et al. Movement Disorder Society-sponsored revision of the Unified Parkinson's Disease Rating Scale (MDS-UPDRS): scale presentation and clinimetric testing results. *Mov Disord* 2008;**23**(15):2129-70.

4. Goetz CG, Stebbins GT, Tilley BC. Calibration of unified Parkinson's disease rating scale scores to Movement Disorder Society-unified Parkinson's disease rating scale scores. *Mov Disord* 2012;**27**(10):1239-42.

**WEB FIGURE LEGENDS**

**Web Figure 1.** Graphical representation of longitudinal associations with intercept and slope. Figure 1.a is top left representing an association with the intercept, figure 1.b is top right representing an association with the slope only and 1.c is bottom left representing an association with both the intercept and slope.

**Web figure 2.** Flow chart to show entry into this study
